# Relative Children’s Lipid Accumulation Product Is a Novel Indicator for Metabolic Syndrome

**DOI:** 10.3389/fendo.2021.645825

**Published:** 2021-05-20

**Authors:** Li Zhang, Zizhe Zhang, Bangxuan Wang, Yongting Yuan, Lili Sun, Huaiquan Gao, Lianguo Fu

**Affiliations:** Department of Child and Adolescent Health, School of Public Health, Bengbu Medical College, Bengbu, China

**Keywords:** metabolic syndrome, relative children’s lipid accumulation product, obesity, children, adolescents

## Abstract

**Background:**

The children’s lipid accumulation product (CLAP) is associated with MS in Chinese children and adolescents. The aim of this study was to develop a more effective indicator, the relative children's lipid accumulation product (RCLAP) was evaluated for correlation with MS and the density of lipid accumulation.

**Methods:**

A stratified cluster sampling method was used to recruit 683 students aged 8–15 years in this study. The presence of MS was defined according to the NCEP-ATP III criteria. The participants’ guardians signed informed consent before the medical examination. This study was approved by the Medical Ethics Committee of the Bengbu Medical College [(2015) No.003] and was conducted in accordance with the Declaration of Helsinki.

**Results:**

The overall prevalence of MS was 4.8% (male 6.6%, female 2.8%). After adjusting for sedentary activity time, relative children's lipid accumulation product per height (RCLAP-H) and relative children's lipid accumulation product per sitting height (RCLAP-SH) significantly increased the risk of MS in girls [OR (95% CI): 96.13 (11.11–831.97) and 96.13 (11.11–831.97), respectively]. After adjusting for ages and moderate-to-vigorous physical activity time, RCLAP-H, and RCLAP-SH significantly increased the risk of MS in boys [OR (95% CI): 171.75 (33.60–878.00) and 133.18 (27.65–641.39), respectively]. The AUCs of RCLAP-H and RCLAP-SH for predicting MS were 0.950, 0.948 in girls, and 0.952, 0.952 in boys, which were higher than BMI, WHtR, Tg/HDL-C, CLAP, and CLAP combining height, sitting height.

**Conclusions:**

The RCLAP-H and RCLAP-SH were more effective indicators for predicting MS than BMI, WHtR, Tg/HDL-C, and CLAP in children and adolescents.

## Introduction

Over the past two decades, there has been a striking increase in the number of people with metabolic syndrome (MS) (1). In 2009, the overall age-standardized estimated prevalence of MS was 21.3% based on the criteria of the revised National Cholesterol Education Program—Third Adult Treatment Panel (NCEP-ATPIII) (2). A meta-analysis showed that MS increased the risk of type 2 diabetes and cardiovascular diseases (CVD) (3). Concomitantly with the increasing prevalence of childhood obesity, the prevalence of metabolic syndrome is rising among children and adolescents (4). According to the International Diabetes Federation (IDF), NCEP-ATPIII, and Chinese Children Metabolic Syndrome Righteousness and Prevention Advice (CHN2012) criteria, the prevalence of MS among Chinese children was 1.8, 2.6, and 2.0%, respectively. In addition, the MS prevalence in children who were overweight and obese was 4.7 and 17.3% based on IDF criteria in 2004–2014, respectively (5). Childhood MS is associated with hypertension, hyperlipidemia, insulin resistance, and type 2 diabetes, which can also lead to cardiometabolic diseases during adulthood. The definition of MS includes the presence of three or more components: central obesity, hypertriglyceridemia, high fasting glucose, low high-density lipoprotein (HDL), and hypertension (6). Many factors may induce MS, including unhealthy eating habits and lack of exercise (7, 8), the etiology and pathogenesis of MS are very complex (9, 10). Thus, more effective indicators to predict MS are very important in children and adolescents.

Some research has reported that body mass index (BMI), waist circumference (WC), abdominal skinfold thickness (AST), Waist-to-height ratio (WHtR), triglycerides (TG), triglycerides-to-HDL-C ratio (Tg/HDL-C), and wrist circumference (WrC) were effectively related with MS (11–17). However, the above indicator is limited to distinguishing adipose tissue from lean mass and showing circulating lipid accumulation. The lipid excess coincides with expansion of visceral adipocytes and elevated blood concentrations of certain lipids, which is referred to as lipid overaccumulation, which could lead to ectopic deposition of lipids in non-adipose tissues, insulin resistance, and other metabolic dysfunctions (18–20). Kahn et al. (18) proposed a new marker, the lipid accumulation product (LAP), which reflects the total lipid accumulation in the body to predict MS in adults. The LAP is a product of waist circumference (WC) and fasting triglycerides (TG) concentration. Studies have shown LAP is a powerful marker for predicting MS and is better than BMI, WC, and WHtR in adults. However, LAP may not directly reflect lipid accumulation in children and adolescents. Zhang et al. (21) developed a novel indicator, the children’s lipid accumulation product (CLAP), associated with MS in Chinese children and adolescents. The CLAP is a product of WC, AST, and TG concentration [CLAP = WC (cm) × AST (mm) × TG (mmol/L) / 100]. They reported that CLAP was an effective indicator associated with MS and was better than BMI and WHtR. Wang et al. (22) showed that CLAP was significantly associated with hypertension in children and adolescents, and can more effectively predict childhood hypertension than WC, WHtR, BMI, AST, and TG. Yuan et al. (23) showed that the CLAP was significantly associated with impaired fasting glucose (IFG) in Chinese boys, and it performed better than WC, WHtR, AST, and TG. From the formula of CLAP, we know that AST refers to the accumulation of skin fat at a point on the abdomen. Multiplying by WC shows the accumulation of whole abdominal fat and then multiplying by TG, to a certain extent, shows the accumulation of body lipid. However, CLAP could not reflect the density of lipid accumulation in body.

It is well known that there are multifarious types of obesity, and different obesity types have different characteristic locations of fat accumulates. Exogenous and endogenous obesity are two types of Childhood obesity (24). The relative children's lipid accumulation product (RCLAP) that was the CLAP at per unit body height, sitting height, and weight, may reflect the density of lipid accumulation among children and adolescents. However, it has been unclear whether the RCLAP were more effective indicators related to MS than CLAP, BMI, and WHtR. The purpose of this study was to develop more effective RCLAP indicators for predicting MS.

## Materials and Methods

### Study Subjects

In this study, a total of 683 students aged 8–15 years were selected from two 9-year schools *via* the stratified cluster sampling methods, including 317 girls (46.4%) aged at (10.98 ± 1.83 years and 366 boys (53.6%) aged at (10.77 ± 1.80 years).

### Measurement

The medical staff who received standardized training measured the participants’ body weight, height, sitting height (SH), Diastolic blood pressure (DBP), Systolic blood pressure (SBP), WC, and AST (21). Venous blood samples (3 ml) were taken by nurses with standardized training after at least 8 h of overnight fasting by the children and adolescents. The enzyme-linked immunoassay method was used to detect HDL-C. Enzymatic methods were used to detected TG and FBG levels.

### Definition of Metabolic Syndrome (MS)

In this study, MS was diagnosed according to the amended NCEP-ATP III criteria (25). High fasting blood glucose (FBG) ≥ 110 mg/dl; abdominal obesity: WC ≥90th age- and sex-specific percentile for Chinese children (26); high blood pressure: SBP and/or DBP ≥90th percentile for gender and age (27); low high-density lipoprotein cholesterol (HDL-C) ≤40 mg/dl; high triglycerides(TG)≥110 mg/dl; when three or more of the five components were present then a diagnosis of MS was made.

### Calculation of the Derivative Variables

Children’s lipid accumulation product (CLAP) = WC (cm) × AST (mm) × TG (mmol/L) / 100; BMI = weight (kg)/height^2^ (m^2^); WHtR = waist circumference (cm)/height (cm); Relative children’s lipid accumulation product per height (RCLAP-H) = WC (cm) × AST (mm) × TG (mmol/L)/height (cm); Relative children’s lipid accumulation product per sitting height (RCLAP-SH) = WC (cm) × AST (mm) × TG (mmol/L)/sitting height (cm); Relative children’s lipid accumulation product per weight (RCLAP-W) = WC (cm) × AST (mm) × TG (mmol/L)/weight (kg).

### Surveys of Behavioral lndexes

We investigated dietary behaviors and physical activities. The healthy dietary behaviors (including breakfast, milk, fruits, nuts, fresh vegetables) and risk dietary behaviors (including fried foods, eating out, carbonated drinks, high-energy snacks, western-style fast food)—each item was assigned 0 points for never, 0.25 points for 1 time per month, 0.5 points for 2 times per month, 2 points for 1–3 times per week, 2 points for 1–3 times per week, 5 points for 4–6 times per week, 7 points for 7 times per week. The total scores of dietary behaviors were defined by the two grades of ≥ the 75th percentile (P75) and <75th percentile (P75) [21–23]. Children’s Leisure Activities Study Survey (CLASS) questionnaire was used to investigate physical activities (21, 28, 29).

### Statistical Analysis

Data were analyzed using SPSS 23.0 software. The data were described using proportion and mean ± standard deviation. The height, siting height, weight, WC, AST, BMI, WHtR, Tg/HDL-C, logarithmic CLAP, RCLAP-H, RCLAP-SH, RCLAP-W, SBP, and DBP were standardized for genders and ages using the Z-score method (Abbreviations of above standardized indexes: SH, SSH, SW, SWC, SAST, SBMI, SWHtR, STg/HDL-C, SlnCLAP, SRCLAP-H, SRCLAP-SH, SRCLAP-W, SSBP, and SDBP, respectively). The t-test, chi-square test, and logistic regression models were used to analyze the associations of SBMI, SWHtR, STg/HDL-C, SlnCLAP, SRCLAP-H, SRCLAP-SH, and SRCLAP-W with MS, respectively. Receiver Operating Characteristic (ROC) curves were used to evaluate the predictive efficiency of above indexes for predicting MS. *P* < 0.05 was considered statistically significant.

## Results

### Demographics 

A total of 683 children aged 8–15 years (366 boys and 317 girls) were included in this study. The overall prevalence of MS was 4.8% (2.8% for girls and 6.6% for boys), as shown in **Table 1**. The results showed that weight, WC, AST, BMI, WHtR, CLAP, RCLAP-H, RCLAP-SH, RCLAP-W, SBP, DBP, Tg/HDL-C, and TG among girls with MS were significantly higher than those without MS; the boys with MS had higher values of height, siting height, weight, WC, AST, BMI, WHtR, CLAP, RCLAP-H, RCLAP-SH, RCLAP-W, SBP, DBP, Tg/HDL-C, and TG compared with those without MS (*P* < 0.05). In contrast, girls and boys with MS had lower values of HDL-C than those without MS, respectively (*P* < 0.05). The prevalence of MS among boys aged 12–15 years was significantly higher than that aged 8–11 years (*P* < 0.05). The proportion of moderate-to-vigorous physical activity time (≥60 min) among boys with MS was significantly lower than those without MS (*P* < 0.05).

**Table 1:** The comparison of anthropometric characteristics, dietary behaviors, physical activities, CLAP, and RCLAP among children with Non-MS and MS.

Variables	Girls (n = 317)			t/χ^2^	*P*	Boys (n = 366)		t/χ^2^	*P*
	Non-MS (97.2%)		MS (2.8%)			Non-MS (93.4%)	MS (6.6%)		
SH	−0.01 ± 0.99		0.32 ± 1.04	−0.98	0.329	−0.03 ± 0.97	0.40 ± 1.17	−2.05	0.041
SSH	−0.01 ± 0.98		0.41 ± 1.19	−1.27	0.204	−0.04 ± 0.96	0.58 ± 1.24	−2.99	0.003
SW	−0.02 ± 0.99		0.83 ± 0.67	−2.59	0.010	−0.10 ± 0.91	1.45 ± 0.91	−8.07	<0.001
SWC	−0.05 ± 0.95		1.74 ± 0.73	−5.61	<0.001	−0.11 ± 0.91	1.58 ± 0.65	−8.90	<0.001
SAST	−0.03 ± 0.98		0.96 ± 0.72	−3.00	0.003	−0.10 ± 0.92	1.48 ± 0.72	−10.22	<0.001
SBMI	−0.02 ± 0.99		0.76 ± 0.52	−2.36	0.019	−0.11 ± 0.91	1.56 ± 0.69	−11.26	<0.001
SWHtR	−0.05 ± 0.95		1.79 ± 0.79	−5.77	<0.001	−0.11 ± 0.92	1.55 ± 0.65	−11.82	<0.001
SlnCLAP	−0.04 ± 0.97		1.43 ± 0.23	−15.42	<0.001	−0.11 ± 0.92	1.60 ± 0.51	−14.91	<0.001
SRCLAP-H	−0.05 ± 0.94		1.85 ± 0.67	−6.01	<0.001	−0.15 ± 0.78	2.20 ± 1.13	−10.03	<0.001
SRCLAP-SH	−0.05 ± 0.94		1.85 ± 0.66	−5.98	<0.001	−0.15 ± 0.79	2.19 ± 1.11	−10.14	<0.001
SRCLAP-W	−0.05 ± 0.96		1.57 ± 0.80	−5.01	<0.001	−0.14 ± 0.80	2.02 ± 1.19	−8.76	<0.001
SSBP	−0.04 ± 0.97		1.32 ± 0.87	−4.18	<0.001	−0.08 ± 0.96	1.09 ± 0.84	−5.84	<0.001
SDBP	−0.04 ± 0.96		1.34 ± 1.02	−4.22	<0.001	−0.06 ± 0.97	0.86 ± 0.91	−4.46	<0.001
HDL-C	1.51 ± 0.29		1.18 ± 0.27	3.32	0.001	1.56 ± 0.30	1.20 ± 0.24	5.81	<0.001
TG	0.94 ± 0.38		1.39 ± 0.27	−3.47	0.001	0.83 ± 0.34	1.50 ± 0.39	−9.24	<0.001
STg/HDL-C	−0.05 ± 0.93		1.86 ± 1.15	−6.02	<0.001	−0.12 ± 0.86	1.71 ± 1.12	−9.83	<0.001
FBG	5.09 ± 0.45		5.26 ± 0.34	−1.08	0.283	5.18 ± 0.42	5.30 ± 0.44	−1.36	0.176
Ages (years)				0.47	0.491			7.06	0.008
8–	187 (96.4)		7 (3.6)			221 (96.1)	9 (3.9)		
12–15	121 (98.4)		2 (1.6)			121 (89.0)	15 (11.0)		
Healthy dietary behaviors				0.13	0.723			0.08	0.769
<*P*_75_	241 (96.8)		8 (3.2)			247 (93.2)	18 (6.8)		
≥*P*_75_	67 (98.5)		1 (1.5)			95 (94.1)	6 (5.9)		
Risk dietary behaviors				0.00	1.00			1.33	0.250
<*P*_75_	256 (97.3)		7 (2.7)			238 (94.4)	14 (5.6)		
≥*P*_75_	52 (96.3)		2 (3.7)			104 (91.2)	10 (8.8)		
Moderate-to-vigorous physical activity time				0.61	0.434			4.46	0.035
<60 min	182 (96.3)		7 (3.7)			166 (90.7)	17 (9.3)		
≥60 min	126 (98.4)		2 (1.6)			176 (96.2)	7 (3.8)		
Sedentary activity time				3.65	0.056			0.38	0.537
<120 min	124 (94.7)		7 (5.3)			179 (94.2)	11 (5.8)		
≥120 min	184 (98.9)		2 (1.1)			163 (92.6)	13 (7.4)		

SH, standardized height; SSH, standardized sitting height; SW, standardized weight; SWC, standardized waist circumference; SAST, standardized abdominal skinfold thickness; SBMI, standardized body mass index; SWHtR, standardized waist-height ratio; SlnCLAP, standardized logarithmic children’s lipid accumulation product; SRCLAP-H, standardized relative children’s lipid accumulation product per height; SRCLAP-SH, standardized relative children’s lipid accumulation product per sitting height; SRCLAP-W, standardized relative children’s lipid accumulation product per weight; SSBP, standardized systolic blood pressure; SDBP, standardized diastolic blood pressure; HDL-C, high-density lipoprotein cholesterol; TG, triacylglycerol; STg/HDL-C, standardized triglycerides-to-HDL-C ratio; FBG, fasting blood glucose.

### The Factors Associated With MS

The results of chi-square test showed that WHtR, CLAP, RCLAP-H, RCLAP-SH, and RCLAP-W were significantly associated with MS among boys and girls (**Table 2**). In girls, after adjusting for sedentary activity time factor, SWHtR ≥1, SBMI ≥1, STg/HDL-C ≥1, SRCLAP-H ≥1, SRCLAP-SH ≥1, and SRCLAP-W ≥1 significantly increased the risk of MS compared with SWHtR <1, SBMI <1, STg/HDL-C <1, SRCLAP-H <1, SRCLAP-SH <1, and SRCLAP-W <1 (OR were 15.79, 3.73, 32.97, 96.13, 96.13, 18.28, respectively). In boys, after adjusting for ages and moderate-to-vigorous physical activity time factors, SWHtR ≥1, SBMI ≥1, STg/HDL-C ≥1, SlnCLAP ≥1, SRCLAP-H ≥1, SRCLAP-SH ≥1, and SRCLAP-W ≥1 significantly increased the risk of MS compared with SWHtR <1, SBMI <1, STg/HDL-C <1, SlnCLAP <1, SRCLAP-H <1, SRCLAP-SH <1, and SRCLAP-W <1 (OR were 37.43, 68.33, 25.70, 105.86, 171.75, 133.18, 50.13, respectively) (**Table 3**).

**Table 2:** The associations between WHtR, BMI, Tg/HDL-C, CLAP, RCLAP, and MS among children.

Variables	Girls (n = 317)		*χ*;^2^	*P*	Boys (n = 366)		*χ* ^2^	*P*
	Non-MS	MS			Non-MS	MS		
SWHtR			18.48	<0.001			58.11	<0.001
<1	258 (99.2)	2 (0.8)			293 (98.3)	5 (1.7)		
≥1	50 (87.7)	7 (12.3)			49 (72.1)	19 (27.9)		
SBMI			2.48	0.115			72.38	<0.001
<1	275 (97.9)	6 (2.1)			298 (98.7)	8 (1.3)		
≥1	33 (91.7)	3 (8.3)			44 (68.8)	20 (31.3)		
STg/HDL-C			31.86	<0.001			71.73	<0.001
<1	277 (89.9)	2 (22.2)			312 (91.2)	7 (29.2)		
≥1	31 (10.1)	7 (77.8)			30 (8.8)	17 (70.8)		
SlnCLAP			44.22	<0.001			90.72	<0.001
<1	268 (100.0)	0 (0.0)			299 (99.3)	2 (0.7)		
≥1	40 (81.6)	9 (18.4)			43 (66.2)	22 (33.8)		
SRCLAP-H			47.67	<0.001			116.98	<0.001
<1	280 (99.6)	1 (0.4)			311 (99.4)	2 (0.6)		
≥1	28 (77.8)	8 (22.2)			31 (58.5)	22 (41.5)		
SRCLAP-SH			47.67	<0.001			111.81	<0.001
<1	280 (99.6)	1 (0.4)			309 (99.4)	2 (0.6)		
≥1	28 (77.8)	8 (22.2)			33 (60.0)	22 (40.0)		
SRCLAP-W			22.78	<0.001			92.46	<0.001
<1	278 (98.9)	3 (1.1)			309 (98.7)	4 (1.3)		
≥1	30 (83.3)	6 (16.7)			33 (62.3)	20 (36.7)		

SWHtR, standardized waist-height ratio; SBMI, standardized body mass index; STg/HDL-C, standardized triglycerides-to-HDL-C ratio; SlnCLAP, standardized logarithmic children’s lipid accumulation product; SRCLAP-H, standardized relative children’s lipid accumulation product per height; SRCLAP-SH, standardized relative children’s lipid accumulation product per sitting height; SRCLAP-W, standardized relative children’s lipid accumulation product per weight.

**Table 3:** The adjusted associations between WHtR, BMI, Tg/HDL-C, CLAP, and RCLAP on MS using logistic regression models.

Variables	*β* (*S*.*E*.)	*Wald*	*P*	*OR* (95% *CI*)
Girls				
SWHtR				
<1	0			1
≥1	2.76 (0.82)	11.24	0.001	15.79 (3.15–79.21)
SBMI				
<1	0			1
≥1	1.32 (0.74)	3.14	0.076	3.73 (0.87–15.95)
STg/HDL-C				
<1	0			1
≥1	3.50 (0.84)	17.35	<0.001	32.97 (6.37–170.80)
SlnCLAP				
<1	0			1
≥1	19.74 (2,358.36)	0.00	0.993	–
SCLAP-H				
<1	0			1
≥1	4.57 (1.10)	17.19	<0.001	96.13 (11.11–831.97)
SCLAP-SH				
<1	0			1
≥1	4.57 (1.10)	17.19	<0.001	96.13 (11.11–831.97)
SCLAP-W				
<1	0			1
≥1	2.91 (0.75)	15.18	<0.001	18.28 (4.24–78.87)
Boys				
SWHtR				
<1	0			1
≥1	3.62 (0.60)	37.09	<0.001	37.43 (11.67–120.10)
SBMI				
<1	0			1
≥1	4.22 (0.67)	40.20	<0.001	68.33 (18.51–252.20)
STg/HDL-C				
<1	0			1
≥1	3.25 (0.51)	40.91	<0.001	25.70 (9.50–59.50)
SlnCLAP				
<1	0			1
≥1	4.66 (0.80)	32.80	<0.001	105.86 (21.99–509.68)
SCLAP-H				
<1	0			1
≥1	5.15 (0.83)	38.21	<0.001	171.75 (33.60–878.00)
SCLAP-SH				
<1	0			1
≥1	4.89 (0.80)	37.20	<0.001	133.18 (27.65–641.39)
SCLAP-W				
<1	0			1
≥1	3.92 (0.60)	42.63	<0.001	50.13 (15.48–162.37)

SWHtR, standardized waist-height ratio; SBMI, standardized body mass index; STg/HDL-C, standardized triglycerides-to-HDL-C ratio; SlnCLAP, standardized logarithmic children’s lipid accumulation product; SRCLAP-H, standardized relative children’s lipid accumulation product per height; SRCLAP-SH, standardized relative children’s lipid accumulation product per sitting height; SRCLAP-W, standardized relative children’s lipid accumulation product per weight.

### The Power for Predicting MS

**Figure 1:**
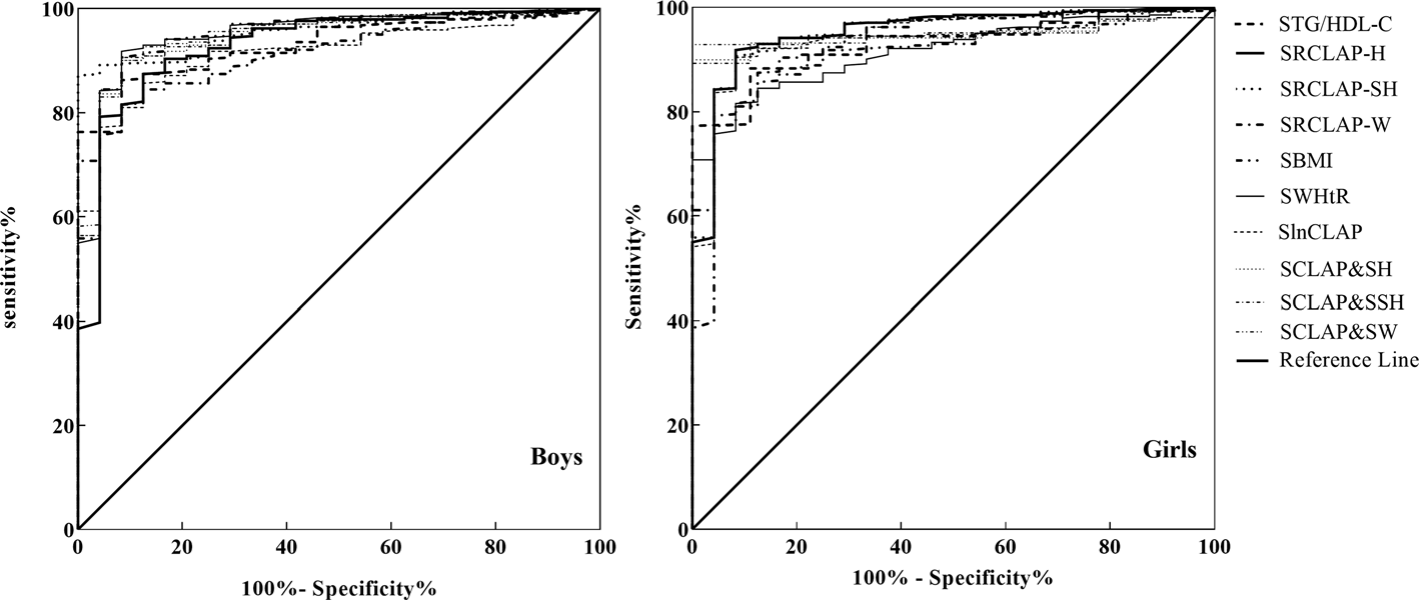
ROC curves of SBMI, SWHtR STg/HDL-C, SlnCLAP SRCLAP-H, SRCLAP-SH, SRCLAP-W, SlnCLAP combining SH, SlnCLAP combining SSH, SlnCLAP combining SW to predict MS among boys and girls. SBMI, standardized body mass index; SWHtR, waist-height ratio; STg/HDL-C, triglycerides-to-HDL-C ratio; SlnCLAP, logarithmic children’s lipid accumulation product; SRCLAP-H, relative children’s lipid accumulation product per height; SRCLAP-SH, relative children’s lipid accumulation product per sitting height; SRCLAP-W, relative children’s lipid accumulation product per weight by sex and age using the z-score method.

As shown in **Table 4** and **Figure 1**, the AUCs of SBMI, SWHtR, STg/HDL-C, SlnCLAP, SRCLAP-H, SRCLAP-SH, SRCLAP-W, SlnCLAP and SH, SlnCLAP and SSH, SlnCLAP and SW for predicting MS among girls were 0.828, 0.925, 0.929, 0.946, 0.950, 0.948, 0.920, 0.947, 0.947, 0.949. The AUCs of above indicators for predicting MS among boys were 0.916, 0.916, 0.931, 0.946, 0.952, 0.952, 0.929, 0.946, 0.946, 0.949, respectively.

**Table 4:** The areas under ROC curves of SBMI, SWHtR, SlnCLAP, SRCLAP-H, SRCLAP-SH, SRCLAP-W, STg/HDL-C, SlnCLAP combining SH, SlnCLAP combining SSH, SlnCLAP combining SW for predicting MS among children.

Variables	AUC	*S*.*E*.	*P*	95% *CI* of AUC
Girls				
SBMI	0.828	0.032	0.001	0.782–0.868
SWHtR	0.925	0.029	<0.001	0.890–0.951
SlnCLAP	0.946	0.014	<0.001	0.916–0.968
SRCLAP-H	0.950	0.014	<0.001	0.920–0.971
SRCLAP-SH	0.948	0.014	<0.001	0.918–0.970
SRCLAP-W	0.920	0.027	<0.001	0.884–0.947
STg/HDL-C	0.929	0.025	<0.001	0.895–0.955
SlnCLAP combining SH	0.947	0.014	<0.001	0.916–0.969
SlnCLAP combining SSH	0.947	0.014	<0.001	0.916–0.969
SlnCLAP combining SW	0.949	0.013	<0.001	0.918–0.969
Boys				
SBMI	0.916	0.020	<0.001	0.883–0.942
SWHtR	0.916	0.019	<0.001	0.883–0.942
SlnCLAP	0.946	0.020	<0.001	0.917–0.967
SRCLAP-H	0.952	0.020	<0.001	0.925–0.972
SRCLAP-SH	0.952	0.020	<0.001	0.925–0.971
SRCLAP-W	0.929	0.027	<0.001	0.898–0.953
STg/HDL-C	0.931	0.017	<0.001	0.900–0.955
SlnCLAP combining SH	0.946	0.020	<0.001	0.917–0.967
SlnCLAP combining SSH	0.946	0.020	<0.001	0.918–0.967
SlnCLAP combining SW	0.949	0.019	<0.001	0.922–0.969

SBMI, standardized body mass index; SWHtR, standardized waist-height ratio; SlnCLAP, standardized logarithmic children’s lipid accumulation product; SRCLAP-H, standardized relative children’s lipid accumulation product per height; SRCLAP-SH, standardized relative children’s lipid accumulation product per sitting height; SRCLAP-W, standardized relative children’s lipid accumulation product per weight; STg/HDL-C, standardized triglycerides-to-HDL-C ratio.

## Discussion

Metabolic syndrome (MS) has become a major public health issue worldwide (30). This study showed that the overall prevalence of MS was 4.8% (males 6.6%, girls 2.8%) in children and adolescents aged 8–15 years. The similar prevalence of MS was reported among Indian studies aged 12–17 years (4.2%) (31) and US adolescents (4.5%) (32). Rodríguezmorán et al. (33) reported that there was higher prevalence of MS among Mexico adolescents (6.5%) and Esmaillzadeh et al. (34) reported that there was 10.1% of Iranian adolescents with MS. The present study showed that prevalence of MS among girls (2.8%) was lower than that in boys (6.6%), which was consistent with the results of most previous studies (25, 35, 36). The reasons might be because the boys had lower levels of moderate-to-vigorous physical activity and higher levels of risk dietary behaviors, so they have a higher prevalence of overweight or obesity among boys than girls. However, other studies had also reported there was no significant difference in the prevalence of MS between sex (9). The current study showed that the prevalence of MS among boys aged 12–15 years was significantly higher than that aged 8–11 years, which was in line with the result of a study by Gooty et al. (37). This may be associated with an increased exposure of risk factors for MS as boys age (38). It was reported that the ability to regulate glucose was progressively lost with age (39). Moreover, in the present study we found that moderate-to-vigorous physical activity time of less than 60 min was a risk factor for MS in boys, which was consistent with previous studies (40, 41). Styne et al. (42) also showed that at least 20 min of vigorous short bursts of physical activity a day, for 3 to 5 days per week can improve metabolic measures in children and adolescents. Physical activity is helpful in improving the lipid profile by increasing HDL concentration and decreasing both LDL and triglycerides concentrations (43).

The additional findings of this cross-sectional study were that the children with MS demonstrated higher BMI, WHtR, Tg/HDL-C, and CLAP levels compared to children without MS, which was in line with previous studies (11, 13, 14, 21). However, these indexes were limited to showing the accumulation of lipids. BMI cannot show an indication of body fat distribution, and it is not only related to fat mass but also related to fat-free mass (44), WHtR was limited to showing the accumulation of lipids in blood circulation, Tg/HDL-C was only showing the accumulation of lipids in blood circulation. CLAP is a better marker to predicting MS than BMI and WHtR in Chinese children and adolescents; however, it only reflects the state of lipid accumulation, not the density of lipid accumulation. Now our results suggested SRCLAP-H and SRCLAP-SH were significantly associated with MS [in girls, the OR values (95% CI) were 96.13 (11.11–831.97) and 96.13 (11.11–831.97), respectively; in boys, the OR value (95% CI) were 171.75 (33.60–878.00) and 133.18 (27.65–641.39), respectively] and the abilities of RCLAP-H and RCLAP-SH for predicting MS were all higher than those of BMI, WHtR, Tg/HDL-C, -CLAP, and CLAP combining height, sitting height. RCLAP-H reflected the lipid accumulation at per unit height which could reflect different metabolic risks based on children’s height; for example, the children with the same CLAP have a greater risk of MS with shorter heights. RCLAP-SH reflected the lipid accumulation at per upper half of body. There have been studies that showed that an upper body or centralized deposition of excess body fat carries an increased risk for obesity-associated metabolic complications (45, 46). In our study population, the effect of SRCLAP-W was not obvious, which may be that WC and AST reflect weight to some extent, so the effect of CLAP divided by weight will be weakened. However, the effect of CLAP combining weight for predicting MS was higher than that of SRCLAP-W, which may be that CLAP combining weight reflects the superposition effect of WC and AST.

Excess lipid material will increasingly be deposited in non-adipose tissues (e.g., liver, kidneys, skeletal muscle, heart, blood vessels, and pancreas) where it may adversely modify cellular metabolism and accelerate apoptosis (47, 48). Commonly adopted predictive indicators of abdominal obesity include WC and related indexes such as the waist-to-height and waist-to-hip ratios (49, 50). Ectopic lipid deposition is difficult to quantify directly in children and adolescents, but an increased RCLAP value may indicate that various tissues or organs have become more vulnerable to injury from lipid overaccumulation. The metabolically obese normal-weight (MONW) (51) individuals who having normal body weight but with obesity, are characterized by the presence of a cluster of cardiovascular risk factors. Janssen (52) proposed that those that also fulfill the criteria for the MS should be classified as MONW. Du et al. (53) showed that LAP and visceral adiposity index (VAI) are effective markers for identifying the Chinese adults with MONW phenotype. We speculate that RCLAP-H or RCLAP-SH may be applicable to identifying MONW in children and adolescents.

The present study has several strengths. The RCLAP at per unit body height, sitting height can reflect the density of lipid accumulation in body, the study demonstrated that the RCLAP-H and RCLAP-SH were more effective indicators for predicting MS than BMI, WHtR, Tg/HDL-C, and CLAP in children and adolescents. However, this study also has some limitations. Firstly, it was a cross-sectional study and the causality between RCLAP and MS cannot be inferred. Secondly, we only studied Chinese children and adolescents, thus the generalizability to other ethnic groups is limited. Finally, the sample size in our study is limited, so we cannot provide a representative cut-off value in different ages and gender for the time being. Therefore, the results need to be confirmed by other studies.

The RCLAP was associated with MS and reflect the density of lipid accumulation among children and adolescents. It is an accurate and simple method for predicting the risk of MS in children and adolescents. Furthermore, we reported that the relative children’s lipid accumulation product per height (RCLAP-H) and relative children’s lipid accumulation product per sitting height (RCLAP-SH) may be more predictive power for MS than BMI, WHtR, Tg/HDL-C, and CLAP.

## Data Availability Statement

The data analyzed in this study is subject to the following licenses/restrictions: This dataset is kept in the School of Public Health, Bengbu Medical College, and can be applied to LF. Requests to access these datasets should be directed to LF, lianguofu@163.com.

## Ethics Statement

The studies involving human participants were reviewed and approved by Medical Ethics Committee of the Bengbu Medical College. Written informed consent to participate in this study was provided by the participants’ legal guardian/next of kin.

## Author Contributions

LZ conceptualized and designed the study, analyzed and interpreted the data, drafted the initial manuscript, reviewed and revised the manuscript. ZZ, BW, YY, and LS collected data, analyzed and interpreted the data, and critically reviewed the manuscript for important intellectual content. HG and LF conceptualized and designed the study, coordinated and supervised data collection, analyzed and interpreted the data, reviewed and revised the manuscript. All authors contributed to the article and approved the submitted version.

## Funding

This project was supported by grants from the National Natural Science Foundation of China (No. 81502823), Outstanding Young Talent Key program of College and University in Anhui Province (No. gxyqZD2017063), 512 Talent Cultivation Plan of Bengbu Medical College (No. by51201204), and Overseas Visiting and Training Project for Outstanding Young Talents in Universities (No. by15200053).

## Conflict of Interest

The authors declare that the research was conducted in the absence of any commercial or financial relationships that could be construed as a potential conflict of interest.
